# Oral Fluid Sampling in Group-Housed Sows: Field Observations

**DOI:** 10.3390/pathogens14090942

**Published:** 2025-09-18

**Authors:** Grzegorz Tarasiuk, Joseph F. Connor, Danyang Zhang, Jeffrey J. Zimmerman

**Affiliations:** 1 College of Veterinary Medicine, Iowa State University, Ames, IA 50011, USA; 2 Carthage Veterinary Service, Ltd., Carthage, IL 62321, USA; 3 College of Liberal Arts and Sciences, Iowa State University, Ames, IA 50011, USA

**Keywords:** swine, oral fluid, surveillance, sampling

## Abstract

Oral fluid sampling is a well-established, non-invasive method for disease surveillance in growing pigs; however, its application in group-housed gestating sows is under-researched. This study (1) characterized sow behaviors associated with oral fluid sampling and (2) documented the transfer of an environmental target into pen-based oral fluid samples. Field observations were conducted on a commercial sow farm in 12 pens of gestating sows sorted by parity (gilts, parity one, and multiparous sows). Sow oral fluid sampling behaviors were quantified by recording interactions with rope samplers using video cameras and then analyzing the recorded footage. All oral fluid sampling attempts were successful. Unlike growing pigs, experience with rope samplers (“training”) did not increase sow participation, but participation in oral fluid collection did increase as sampling time increased. The transfer of environmental components into oral fluid samples was demonstrated by introducing a fluorescent tracer into the pen and then detecting specific fluorescence in the samples (8 of 12 pens). These findings support the implementation of oral fluid sampling in group-housed sows and provide practical recommendations for optimizing surveillance protocols, including extended sampling times and use of at least two ropes per pen.

## 1. Introduction

Sow housing has undergone significant changes in recent decades. As reviewed by McGlone (2013) [1], gestating sows were historically held in open pens, lots, or pastures, but by the late 1980s, most were housed in individual stalls to facilitate population management and better control of feed intake [2]. More recently, animal welfare concerns have prompted a return to group housing with the objective of providing sows more space to move and express natural behaviors [3]. Thus, 2013 European Union legislation mandated housing pregnant sows in groups from four weeks post-insemination until one week before farrowing [3]. Elsewhere, producers are also increasingly utilizing pen gestation, e.g., Tarasiuk et al. (2024a) [4] reported that 65 of 99 U.S. producers with breeding herds used open pens for gestating sows. Thus, the swine industry has come full circle, from group housing to individual animal housing and back to group housing.

Separate from the issue of housing but consequential in its own right, the health of the sow herd is increasingly recognized for its post-weaning impact on pig health and mortality. Magalhães et al. (2024) [5] noted that “... the sow farm plays a major role in the downstream survivability of growing pigs.” Even so, implementing effective sow herd external and internal biosecurity has been problematic because of the ongoing activities related to managing the people, animals, and finances of a complex enterprise. Regardless, oversight of sow herd health is crucial because of its potentially system-wide financial and health impacts. This underlines the need for a practical, diagnostically sensitive, yet cost-effective breeding herd surveillance plan.

Oral fluid sampling is a non-invasive approach to herd-level disease surveillance that is widely used in growing pig populations. As reviewed by Munguía-Ramírez et al. (2022) [6], the detection of agent-specific nucleic acid and/or antibody in oral fluids has been reported for essentially all common pathogens of swine. However, the use of oral fluids remains underexplored in group-housed sows. Therefore, two objectives were addressed in this study. Our first objective was to quantify behaviors associated with oral fluid sampling in group-housed gestating sows, specifically the proportion of sows in a pen that interacted with rope samplers (“participation”), the time participants spent interacting with rope samplers (“contact time), sow behavior when two ropes were placed in the pen (“choice”), and the effect of parity and experience with rope samplers (“training”) on these parameters. The second objective was to determine whether an analytical target introduced into a pen environment would be transferred by the sows into a pen-based oral fluid sample [7]. Ultimately, the goal of this work was to provide data that will assist producers and veterinarians in implementing oral fluid-based surveillance in breeding herds and enhancing disease monitoring in modern swine production systems.

## 2. Materials and Methods

### 2.1. Experimental Design

This study was conducted on a commercial breed-to-wean sow farm (6500 head) in Illinois, USA. The farm was equipped with modern installations for receiving, housing, and managing breeding herd populations, i.e., quarantine, gilt development, pen gestation, and farrowing facilities. The gestation pens housing the animals in this study were fully slatted and held 56 mid-gestation sows segregated by parity group: gilts (first pregnancy), parity one sows (second pregnancy), or multiparous sows. Each pen was equipped with four individual sow automatic feeding stations (Gestal Select, St. Lambert de Lauzon, QC, Canada) and nipple drinkers.

The project was conducted by collecting oral fluids (2 ropes per pen) for 4 consecutive days from 12 pens of gestating sows: 3 pens of gilts, 3 pens of parity one sows, and 6 pens of multiparous sows. For Objective 1 (characterization of sow oral fluid sampling behaviors), 30 of the 56 females in each pen were individually marked for visual identification (Ideal^®^ Prima^®^ Spray-On, Neogen Corp., Lansing, MI, USA), and their interactions with rope samplers were tabulated and analyzed using video recordings taken during sampling on days 1, 2, and 3. For Objective 2 (detection of an environmental target in an oral fluid sample), a solution (20 mL) containing a fluorescent tracer (food coloring) was poured onto the floor in the center of the pen immediately before oral fluid collection on day 4. The level of fluorescence (relative fluorescence units, RFU) in day 4 (treatment) oral fluid samples was compared to day 3 (background) oral fluid samples from the same pen to determine whether the tracer was transferred to the samples.

### 2.2. Oral Fluid Collection

Oral fluid collections were performed as described elsewhere [7,8]. In brief, oral fluid samples were collected using 100% cotton rope (1.3 cm 3-strand twisted 100% cotton rope, Skydog Rigging Equipment, Lake in the Hills, IL, USA). Each sampling (90 min) was performed by placing 2 ropes on the walkway side of the pen, i.e., one rope ~150 cm (~4.9 feet) from each corner, with the 2 ends of each rope suspended at the sows’ shoulder height. At the end of the collection period, oral fluid was collected by placing the rope in a plastic bag and hand-squeezing to release the fluid. For Objective 1, the oral fluid that accumulated in the bottom of the 2 plastic bags (one for each rope) was poured into a 50 mL tube (Southern Labware^®^, Cumming, GA, USA), and the aggregate volume recorded. For Objective 2, the oral fluid samples collected on days 3 and 4 were placed on wet ice and then processed in the laboratory prior to fluorometric testing, as described below.

### 2.3. Objective 1: Sow Behavior During Oral Fluid Collection

Objective 1 required quantifying sow oral fluid collection behaviors, i.e., participation in oral fluid sampling, rope contact time, and rope selection (choice) when two ropes were available in the pen. On 3 consecutive days (days 1, 2, 3) collections (90 min) in the 12 pens were recorded using video cameras (GoPro^®^ 7 cameras, GoPro Inc., San Mateo, CA, USA), which were strategically placed to visualize the interactions of the sows with the ropes. In each pen, 30 sows were clearly numbered (Ideal^®^ Prima^®^ Spray-On) to permit individual sow identification in the video recordings.

After completion of the oral fluid collections, video recordings were viewed and behaviors tabulated for each marked sow. In evaluating the video recordings, “contact” was defined as an individually identified sow clearly taking the rope into her mouth. A “contact count” was defined as ≥1 rope contacts by a sow in a one-minute interval of the observation period, i.e., multiple contacts in the same minute by the same sow were counted as “1”. “Contact time” for an individual sow was defined as the number of contact counts in a defined observation period (30, 60, or 90 min). Thus, there would be a maximum of 30 contact counts in a 30 min observation period, etc. “Participation” was defined as the proportion of marked sows in a pen with ≥1 rope contacts during the observation period (30, 60, or 90 min). “Interval-in-time participation” was defined as the proportion of the 56 sows in a pen, i.e., both marked and unmarked sows, with rope contacts during a specific one-minute interval (minute 1, 5, 10, 15, etc.) of the sampling period. “Rope choice” was used to describe sow rope selection given that 2 ropes were provided in each pen in this study, i.e., sows interacted either with one or both ropes.

### 2.4. Objective 2: Transfer of an Environmental Target into an Oral Fluid Sample

Objective 2 addressed the question of whether a target placed in the pen environment would be transferred into an oral fluid sample. The real question is whether a bacterial or viral pathogen present in the pen environment would be transferred and detected in an oral fluid sample. However, for practical and ethical considerations, red food coloring (McCormick & Co., Inc., Hunt Valley, MD, USA) containing 2 fluorescent compounds (Allura Red AC and erythrosine) was used as a surrogate [9,10,11,12]. Thus, this approach allowed for quantifiable results of target transfer without affecting the health of the animals or the productivity of the farm.

To conduct the experiment, a tracer stock solution was prepared by adding 500 mL of sugar solution (300 g of white sugar in 300 mL of water) to 500 mL of red food coloring. Immediately prior to collecting oral fluid samples on day 4, the tracer solution (50 mL) was poured on the floor in the approximate center of each pen, and then 2 oral fluid collection ropes were suspended in the pens for 90 min. The criterion for documenting the transfer of the tracer from the environment into oral fluid samples was a significant increase in fluorescence in samples collected on day 4 when compared to fluorescence levels in samples collected on day 3 from the same pen. That is, fluorescence levels in pre-treatment (day 3) oral fluid samples were used to establish a baseline for comparison and account for any naturally fluorescing substances in the pen environment, e.g., urine, feces, feed mycotoxins, etc.

The fluorescence testing procedure has been fully described elsewhere [7]. In brief, day 3 (pre-treatment) and 4 (post-treatment) oral fluid samples were centrifuged (3300× *g* for 90 min at 22 °C). Thereafter, the supernatant was tested for fluorescence using a SpectraMax^®^ i3x Multi-Mode Microplate Reader (Molecular Devices LLC, San Jose, CA, USA) and control software (SoftMax^®^ Pro 6.5 Molecular Devices LLC), with the response reported in relative fluorescence units (RFUs). Initially, the optimized excitation and emission wavelengths of the red food coloring were established as 530 nm and 570 nm, respectively, using the control software spectral optimization function.

To perform the testing, oral fluid supernatant samples were vortexed and then tested in duplicate by pipetting 100 μL of sample into one well in each of 2 96-well, black-walled, clear-bottom plates (Thermo Fisher Scientific™ Nunc, Waltham, MA, USA). Concurrently with the field samples, a standard curve based on the stock solution was run in duplicate on each plate (blank, 1:100 and then 2-fold dilutions through 1:204,800). To account for background fluorescence and for the effect of the oral fluid matrix on fluorescence readings, oral fluid was used as the diluent for the standard curve dilutions. The oral fluid diluent was created by pooling 3 mL of the pretreatment (day 3) oral fluid samples collected from each pen (n = 12).

### 2.5. Data Analysis

Descriptive statistics for the oral fluid volume data, i.e., mean and 95% confidence intervals, were calculated in Microsoft Excel^®^ (Redmond, WA, USA). Analyses of data on contact time, sow participation, rope choice, interval-in-time participation, and the transfer of environmental targets into oral fluid samples, were performed in R 4.4.0 (R core team, 2023) [13]. Mean contact time was analyzed using a mixed-effects linear model (package lme4, v1.1-35.1) [14]. Because the proportion of sows that interacted with the rope was bounded by 0 and 1, cumulative participation and rope choice were analyzed using a mixed-effects generalized linear model with a binominal link (package lme4, v1.1-35.1) [14]. Statistical models for analyzing cumulative sow participation, contact time, and rope choice used pen ID as a random effect and parity group (gilts, parity one sows, multiparous sows), sampling day (1, 2, 3) and time (30, 60, 90 min) as categorical fixed effects. Time (minutes) was classified as a categorical factor because of the non-linear relationship between time and behavioral outcomes observed in the data. The analysis of interval-in-time participation used pen ID as a random effect, with parity group and sampling day as categorical fixed effects. All models except for rope choice were fitted without interactions because no statistical significance (*p* > 0.05) was detected among fixed effects. The effect of previous experience (training) and parity group on sow participation, contact time, rope choice, and interval-in-time participation for data collected on sampling days (1, 2, 3), was evaluated using a type-III analysis of variance (ANOVA) on the fitted models (package car, v3.1-2) [15]. Post hoc pairwise comparisons were conducted when the overall Chi-square test was significant (emmeans package, v1.10.0) [16]. Thereafter, simulations were conducted using bootstrap methods to provide predictions more broadly applicable to the field. Specifically, predicted mean percentages of sow participation, contact time, choice (one or both ropes), and 95% prediction intervals were derived from 10,000 bootstrap simulations for each factor combination of interest using the fitted models (merTools package, v0.6.2) [17].

Analysis of the fluorescence data was based on the mean lnRFU calculated from the technical duplicates of each pen-based oral fluid sample. Because the samples were collected at the same time points from animals housed under identical conditions, receiver operating characteristic (ROC) analysis (pROC package v1.18.5) [18] was performed on the aggregate data, i.e., test results from all pens and all parity groups, to identify samples positive for the fluorescent tracer. ROC analysis is based on the analysis of data from samples of known status. Therefore, the pre- and post-treatment samples, i.e., samples collected on day 3 and day 4, were classified as known negative and positives, respectively.

Using Youdin’s J as the optimum cutoff, the number of positive samples and their mean lnRFU (95% confidence intervals) were calculated for each parity group. For positive samples, lnRFU responses were also expressed as a percentage (%) of the concentration of the fluorescent tracer in the stock solution poured on the floor of the pen. This calculation was based on the standard curve run on each plate and the inverse.predict function of R chemCal() package v0.2.3 [19].

## 3. Results

In the three gilt pens, the mean volumes of oral fluid collected by pooling the two ropes placed in each pen were 43 mL (95% CI 34, 53), 31 mL (95% CI 21, 40), 29 mL (95% CI 20, 37), and 35 mL (95% CI 28, 42) on days 1, 2, 3, and 4, respectively. For the same sampling days, parity one sows produced 41 mL (95% CI 34, 49), 35 mL (95% CI 29, 41), 29 mL (95% CI 21, 37), and 37 mL (95% CI 30, 43), and multiparous sows produced 29 mL (95% CI 22, 36), 32 mL (95% CI 24, 39), 27 mL (95% CI 21, 34), and 30 mL (95% CI 26, 34). A mixed-effects linear model (package lme4, v1.1-35.1) [14] fitted to the data showed a significant difference in volume among production groups (*p* < 0.01), sampling day (*p* < 0.001), and [production group × sampling day] (*p* < 0.01). Regardless, in all production groups and sampling days, the volume of oral fluid collected was more than adequate for diagnostic testing.

Table 1 reports the percentages of marked sows that participated in oral fluid sampling by parity group (gilts, parity one sows, multiparous sows), sampling day (1, 2, 3), and sampling time (30, 60, 90 min). All post hoc pairwise comparison *p*-values were adjusted using Tukey’s method to control a familywise error rate of 0.05. Predicted values and predicted intervals derived from simulations are likewise listed in Table 1 to more broadly extrapolate the results to the field. Significant differences in sow participation were detected among parity groups (*p* = 0.040, type-III ANOVA) and sampling time (*p* < 0.001, type-III ANOVA). In all parity groups, sampling time had a significant (positive) effect on sow participation (*p* < 0.001). Post hoc pairwise comparisons among parity groups found a difference in participation between gilts and sows (*p* = 0.033), but not between gilts and parity one sows (*p* = 0.523) or parity one sows and multiparous sows (*p* = 0.426). No training effect was observed in any parity group, i.e., sow participation did not increase by sampling day (*p* = 0.585). Overall, mean participation (%) was higher in younger animals but increased in all parity groups as sampling time increased.

Table 2 reports the mean contact time (minutes) with rope samplers by parity group (gilts, parity one sows, multiparous sows), sampling day (0, 1, 2), and sampling time (30, 60, 90 min). Differences in contact time were observed among parity groups (*p* = 0.017, type-III ANOVA), sampling days (*p* = 0.013, type-III ANOVA), and sampling time (*p* < 0.001, type-III ANOVA). Post hoc pairwise comparisons among parity groups detected a difference in mean contact time between parity one and multiparous sows (*p* = 0.048). Post hoc pairwise comparisons likewise showed a difference in mean contact time between days 0 and 2 (*p* = 0.016) but not between days 0 and 1 (*p* = 0.783) or days 1 and 2 (*p* = 0.085) among all parity groups. Overall, mean contact time (minutes) was higher in younger animals but increased in all parity groups as sampling time increased.

Table 3 reports rope choice (interaction with one vs. both ropes) expressed as the percentage of sows that contacted both rope samplers in the pen. Results are given by parity group (gilts, parity one sows, multiparous sows), sampling day (0, 1, and 2), and sampling time (30, 60, 90 min). Rope choice differed significantly by parity group (*p* = 0.039, type-III ANOVA), sampling time (*p* = 0.049, type-III ANOVA), and (parity group × sampling day) (*p* = 0.019, type-III ANOVA). Post hoc pairwise comparisons detected significant differences in rope choice between gilts and multiparous sows (*p* < 0.001), as well as between gilts and parity one sows (*p* = 0.027). Within parity groups, post hoc pairwise comparisons among sampling days only detected differences in rope choice in multiparous sows between days 0 and 1 (*p* = 0.010) and days 0 and 2 (*p* = 0.007). The percentage of animals that contacted both ropes declined as parity increased, but increased in all parity groups as sampling time increased.

**Figure 1:**
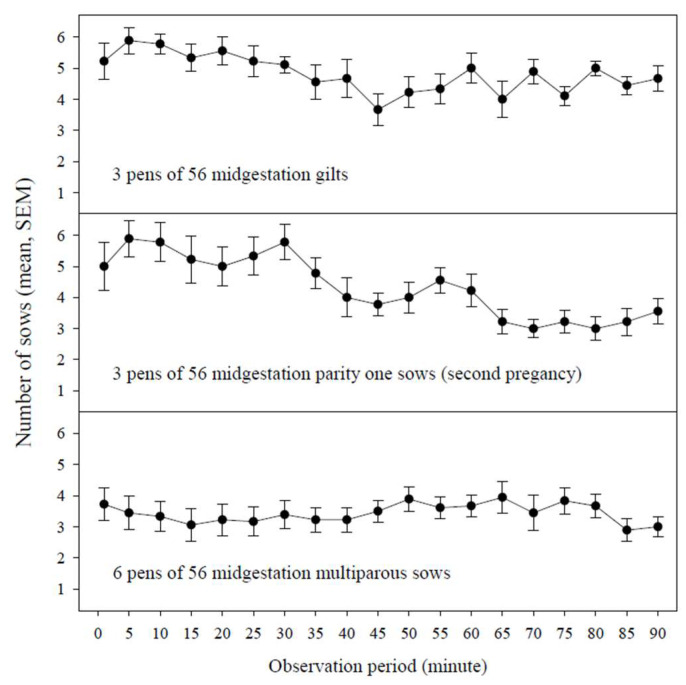
Interval-in-time participation, i.e., the number of sows (mean, SEM) in a pen of 56 animals with rope contacts during a specified observation minute (1, 5, 10, 15, etc.) by parity group (gilts, parity one sows, multiparous sows).

Figure 1 provides interval-in-time participation, i.e., the number of sows (mean, SEM) in a pen of 56 animals with rope contacts during a specified observation minute (1, 5, 10, 15, etc.) by parity group (gilts, parity one sows, multiparous sows). No significant difference in interval-in-time participation was detected among parity groups, sampling days, or sampling times (30, 60, 90 min) (*p* ≥ 0.50, type-III ANOVA). Therefore, interval-in-time participation is presented as the mean of three observations in three pens of gilts, three pens of parity one sows, and six pens of multiparous sows.

The pre- and post-treatment fluorescence (lnRFU) responses (mean, 95% confidence interval) and ROC cutoffs are presented in Table 4 (Steps 1 and 2) based on the analysis of the aggregate data, i.e., test results from all pens and all parity groups. Based on the ROC analysis, Youdin’s J, the optimum cutoff to discriminate between samples containing the fluorescent tracer, was 10.69 lnRFU.

All pre-treatment samples were below the ROC cutoff (Table 4, Step 3a). Among the post-treatment samples, 8 of 12 were above the cutoff, i.e., the fluorescent tracer was detected (Table 4, Step 3b). Based on an analysis of the standard curves run on each plate, the concentration of red food coloring in the eight positive oral fluid samples was estimated as 0.003% (95% CI 0.005%, 0.001%) of the stock solution placed on the floor of each pen.

## 4. Discussion

A major shift toward the use of oral fluid specimens in diagnostic medicine began with the U.S. Food and Drug Administration’s approval of the first oral fluid sampling device for HIV antibody testing in 1994 [20]. Thereafter, technological developments produced oral fluid-based assays for a variety of infectious and non-infectious analytes in humans and enabled the widespread use of oral fluid in SARS-CoV-2 testing during the 2020–2023 pandemic [21,22,23,24,25].

In veterinary medicine, the routine use of oral fluid samples was initiated by a 2006 report that described the detection of porcine reproductive and respiratory syndrome virus (PRRSV) RNA and antibody in oral fluids from pigs and postulated that oral fluids could provide “... an efficient, cost-effective, and practical method for the surveillance of PRRSV and other pathogens ....” [26]. Mirroring the developments in human diagnostics, oral fluid antibody and/or nucleic acid assays have been described for all major swine pathogens [6] and oral fluid has become the most common disease surveillance specimen submitted for testing at swine-focus U.S. veterinary diagnostic laboratories [27,28,29,30]. In addition, swine oral fluids have been shown to contain detectable levels of hormones [31], stress biomarkers [32], and antimicrobial compounds [33].

The routine collection of oral fluids from individual animals or from pens of pigs is possible because it reflects normal pig behavior [34,35]. That is, pigs typically investigate novel objects (in this case, a rope introduced into the pen) by tasting or chewing. Pigs also observe and learn from other pigs [36]; thus, pigs playing with a rope will prompt similar behavior in other pigs. While oral fluid sampling reflects normal behavior, specific factors affect pig participation in oral fluid sampling in production settings: (1) prior experience with rope samplers (training), (2) the length of time pigs are given to access the rope (sampling time), and (3) the number of pigs in the pen (pen size).

The effect of training, i.e., prior experience with rope samplers, on pig participation in oral fluid sampling was first described by White et al. (2014) [37]. In commercial farms holding 25 to 28 growing pigs per pen, participation in oral fluid collections (one rope per pen, 30 min sampling time) increased from 54.4% in pens holding pigs with no prior experience to 75.5% in pens of trained pigs. Similarly, in finishing pens holding ~125 pigs (one rope per pen, 60 min sampling time), Tarasiuk et al. (2024c) [8] reported that participation increased from 35% in pens of untrained pigs to 56% in pens with trained pigs.

The effect of sampling time on pig participation in oral fluid sampling has likewise been investigated by several researchers. White et al. (2014) [37] and Graage et al. (2019) [38] collected oral fluids for 30 min in pens holding 25 to 28 and 16 to 30 growing pigs, respectively. Seddon et al. (2012) [39] collected oral fluids for 60 min in pens holding 17 to 24 growing pigs. In trained pigs, the results were similar across these independent studies, i.e., ~70% of the pigs participated in oral fluid collection over a 30 to 60 min collection period.

In a larger study, Tarasiuk et al. (2024c) [8] measured the effect of sampling time (up to 90 min) in finishing pigs and over a range of pen sizes (30, 90, and 120 pigs per pen). Regardless of pen size, as sampling time increased, participation increased. For example, in pens of 90 pigs, 37% of the population participated in oral fluid collection at 30 min vs. 57% at 90 min. The effect of pen size was more complicated. When evaluated as percentage of participation in oral fluid sampling, Tarasiuk et al. (2024c) [8] found that each additional 10 pigs in a pen reduced participation by 2.8% in a 90 min sampling period. However, when expressed in terms of pig numbers, participation rates of 64%, 47%, and 38% of pigs in pens of 30, 90, and 120 pigs in a 60 min sampling period resulted in ~19, ~42, and ~46 pigs, respectively, contributing to a pen-based oral fluid sample [8]. Thus, larger pen sizes provide an advantage in terms of the number of pigs contributing to the sample. Based on these data, Tarasiuk et al. (2024c) [8] recommended sampling for ≥60 min to maximize pig participation and collect an oral fluid sample most representative of the status of the pigs in the pen.

In production settings, most oral fluid sampling is carried out post-weaning through finishing, and the majority of the research has also focused on this age range. Even so, previous work showed that oral fluids can be collected from adult swine. Kittawornrat et al. (2013) [34] and Pepin et al. (2015) [40] both described the use of oral fluid sampling for monitoring PRRSV in individually-housed boars. Pierdon et al. (2016) [41] researched oral fluid collection in group-housed sows in two pens (175 sows in each pen) equipped with electronic sow feeding system. Oral fluids were collected using one rope per pen for 55 min once a week for 3 consecutive weeks. On average, 19.9 sows interacted with the rope and sampling days did not impact participation. Fablet et al. (2017) [42] and Pol et al. (2017) [43] collected oral fluids in 35 herds in Brittany, France using one rope per pen (45 min sampling time) and reported the successful sample collection from 82 of 137 pen with average sow participation of 46.2%.

The results of the present study corroborated previous publications and expanded our understanding of the oral fluid sampling process in sows. In contrast to observations in growing/finishing pigs, no training effect was detected in sows. That is, regardless of parity group, experience with rope samplers did not result in increased sow participation by sampling day (*p* = 0.585). Separate from the issue of training but relevant to this discussion, participation declined by parity group age, e.g., participation in gilts (40–43%) differed (*p* = 0.033) from participation in multiparous sows (25–29%). As reviewed by Blois-Heulin et al. (2015) [44], play behavior is important in young animals but, for the most part, is lost in adulthood. Therefore, we hypothesize that the robust success of oral fluid sampling in sows is due to the hardwired instinct to explore novel objects by chewing, whereas the absence of a training effect across all sow groups and declining participation in older sows is an expression of adults’ decreasing inclination to engage in play behavior.

## 5. Conclusions

Oral fluids can be routinely collected from group-housed gestating sows. Distinct from growing/finishing pigs, however, sampling time in group-housed sows should be 60 to 90 min in order to maximize participation. In addition, we recommend placing two sampling ropes per pen to provide adequate access and reduce competition for a limited “resource”. To reduce costs, these two samples from the same pen should be pooled prior to testing.

This study focused on behavior rather than diagnostic testing, but our results confirm the observation that, in the process exploring their environment, pigs can transfer detectable levels of diagnostic targets from the pen into the oral fluid sample [7]. In this case, a fluorescent tracer (20 mL) placed on the floor was detected in oral fluids from 8 of 12 pens. This explains the presence of non-oral targets, e.g., PEDV, in oral fluids and justifies their use for the surveillance of non-oral pathogens [45].

## Figures and Tables

**Table 1:** Sow participation (%) by sampling time (30, 60, and 90 min) based on field observations ^1^ and simulations ^2^.

Sow Parity Group	Sampling Day	Data Source	Mean Sow Participation (95% CI or 95% PI)		
			30 min	60 min	90 min
Gilts	Day 1	Field data *Predicted*	40% (10, 70) 42% (9, 85)	44% (18, 71) 53% (13, 89)	53% (39, 68) 58% (15, 91)
	Day 2	Field data *Predicted*	43% (29, 58) 43% (9, 85)	59% (22, 96) 54% (13, 90)	60% (27, 93) 59% (15, 92)
	Day 3	Field data *Predicted*	42% (13, 71) 41% (8, 84)	56% (46, 65) 51% (12, 89)	58% (53, 63) 56% (15, 90)
Parity one sows	Day 1	Field data *Predicted*	40% (11, 69) 34% (6, 79)	42% (11, 74) 44% (10, 85)	47% (11, 83) 50% (12, 88)
	Day 2	Field data *Predicted*	36% (10, 61) 35% (7, 80)	44% (21, 68) 45% (10, 85)	49% (14, 83) 50% (12, 88)
	Day 3	Field data *Predicted*	33% (25, 42) 33% (6, 79)	42% (21, 63) 43% (9, 85)	50% (14, 86) 48% (11, 87)
Multiparous sows	Day 1	Field data *Predicted*	29% (14, 45) 27% (5, 73)	39% (26, 53) 36% (8, 80)	44% (30, 59) 41% (9, 83)
	Day 2	Field data *Predicted*	25% (15, 35) 28% (5, 73)	38% (22, 54) 37% (8, 80)	43% (22, 65) 42% (10, 84)
	Day 3	Field data *Predicted*	26% (15, 38) 26% (5, 72)	33% (18, 48) 35% (7, 79)	39% (24, 55) 40% (9, 82)

^1^ Field data presented as mean percentage of sow participation (%) and 95% confidence intervals (CI). Significant differences in sow participation were detected among parity groups (*p* = 0.040, type-III ANOVA) and sampling time (*p* < 0.001, type-III ANOVA). In all parity groups, sampling time had a significant (positive) effect on cumulative sow participation (*p* < 0.001). Post hoc pairwise comparisons among parity groups found a difference in cumulative participation between gilts and sows (*p* = 0.033), but not between gilts and parity one sows (*p* = 0.523) or parity one sows and multiparous sows (*p* = 0.426). No training effect was observed for any parity group, i.e., sow participation did not increase by sampling day (*p* = 0.5849). ^2^ Predicted sow participation given as mean (%) and 95% prediction intervals (PI) based on 10,000 bootstrap simulations of the fitted models (merTools package, v0.6.2).

**Table 2:** Rope contact time (minutes) by sampling time (30, 60, and 90 min) based on field observations ^1^ and simulations ^2^.

Sow Parity Group	Sampling Day	Data Source	Mean Contact Time (95% CI or 95% PI)		
			30 min	60 min	90 min
Gilts	Day 1	Field data Predicted	9 min (5, 14) 9 min (4, 15)	15 min (13, 16) 12 min (7, 18)	15 min (11, 19) 14 min (9, 19)
	Day 2	Field data Predicted	7 min (6, 9) 9 min (4, 14)	10 min (8, 12) 12 min (7, 17)	14 min (11, 17) 14 min (8, 19)
	Day 3	Field data Predicted	9 min (3, 15) 8 min (3, 13)	10 min (9, 11) 11 min (5, 16)	13 min (8, 17) 13 min (7, 18)
Parity one sows	Day 1	Field data Predicted	6 min (1, 11) 7 min (2, 13)	11 min (1, 21) 10 min (5, 15)	13 min (4, 23) 12 min (7, 17)
	Day 2	Field data Predicted	7 min (2, 11) 7 min (2, 12)	10 min (8, 11) 10 min (4, 15)	11 min (8, 15) 12 min (6, 17)
	Day 3	Field data Predicted	6 min (3, 8) 6 min (0, 11)	9 min (7, 11) 8 min (3, 14)	9 min (5, 13) 10 min (5, 16)
Multiparous sows	Day 1	Field data Predicted	6 min (3, 10) 7 min (1, 12)	9 min (7, 11) 9 min (4, 15)	10 min (8, 12) 11 min (6, 17)
	Day 2	Field data Predicted	7 min (6, 8) 6 min (1, 12)	9 min (5, 14) 9 min (4, 14)	12 min (5, 19) 11 min (6, 16)
	Day 3	Field data Predicted	6 min (5, 8) 5 min (0, 10)	8 min (5, 10) 8 min (3, 13)	9 min (7, 12) 10 min (5, 15)

^1^ Field data presented as mean rope contact time in minutes (min) and 95% confidence intervals. Differences in contact time were observed among parity groups (*p* = 0.017, type-III ANOVA), sampling days (*p* = 0.013, type-III ANOVA), and sampling time (*p* < 0.001, type-III ANOVA). Post hoc pairwise comparisons among parity groups detected a difference in mean contact time between parity one and multiparous sows (*p* = 0.048). Post hoc pairwise comparisons showed a difference in mean contact time between days 0 and 2 (*p* = 0.016) but not between days 0 and 1 (*p* = 0.783) or days 1 and 2 (*p* = 0.085) among all parity groups. ^2^ Predicted contact time in minutes (min) given as mean values and 95% prediction intervals (PI) based on 10,000 bootstrap simulations of the fitted models (merTools package, v0.6.2).

**Table 3:** Sows (%) that contacted both ropes by sampling time (30, 60, 90 min) based on field observations ^1^ and simulations ^2^.

Sow Parity Group	Sampling	Data Source	Both Ropes Contacted (95% CI or 95% PI)		
			30 min	60 min	90 min
Gilts	Day 1	Field data Predicted	23% (2, 45) 22% (4, 68)	30% (5, 55) 30% (5, 77)	33% (11, 55) 34% (6, 80)
	Day 2	Field data Predicted	22% (17, 27) 24% (4, 71)	36% (18, 53) 34% (6, 80)	40% (23, 57) 39% (8, 84)
	Day 3	Field data Predicted	28% (9, 47) 27% (5, 74)	34% (8, 61) 35% (7, 81)	41% (29, 54) 41% (8, 84)
Parity one sows	Day 1	Field data Predicted	14% (2, 27) 15% (2, 59)	19% (2, 36) 20% (3, 66)	28% (0, 57) 26% (4, 73)
	Day 2	Field data Predicted	14% (10, 19) 12% (2, 53)	18% (8, 27) 17% (3, 62)	22% (10, 35) 24% (4, 71)
	Day 3	Field data Predicted	11% (6, 16) 12% (2, 52)	17% (8, 25) 15% (2, 59)	22% (3, 41) 22% (3, 69)
Multiparous sows	Day 1	Field data Predicted	11% (2, 19) 10% (2, 48)	21% (10, 32) 19% (3, 65)	24% (13, 36) 24% (4, 71)
	Day 2	Field data Predicted	7% (2, 11) 6% (1, 35)	12% (5, 19) 12% (2, 52)	18% (7, 30) 17% (3, 60)
	Day 3	Field data Predicted	7% (5, 10) 7% (1, 36)	12% (6, 19) 12% (2, 51)	17% (3, 30) 16% (3, 59)

^1^ Field data (%) presented as mean percentage (%) of sows contacting both ropes and 95% confidence intervals. Sow choice differed significantly by parity group (*p* = 0.039, type-III ANOVA), sampling time (*p* = 0.049, type-III ANOVA), and (parity group × sampling day) (*p* = 0.019, type-III ANOVA). Post hoc pairwise comparisons detected significant differences in rope choice between gilts and multiparous sows (*p* < 0.001), as well as gilts and parity one sows (*p* = 0.027). Within parity groups, post hoc pairwise comparisons among sampling days only detected differences in rope choice in multiparous sows between days 0 and 1 (*p* = 0.010) and days 0 and 2 (*p* = 0.007). ^2^ Predicted (%) presented as mean percentage (%) of sows contacting both ropes and 95% prediction intervals (PI) based on 10,000 bootstrap simulations of the fitted models (merTools package, v0.6.2).

**Table 4:** Detection of fluorescence ^1^ in pen-based oral fluid samples following placement of a fluorescent tracer ^2^ (50 mL) on the pen floor.

Step-Wise Analysis	1st Gestation (3 pens)	2nd Gestation (3 pens)	Multiparous Sows (6 pens)
1. Pre-treatment (day 3 sample) lnRFU means (95% CI) for each production group	10.61 (10.45,10.78)	10.38 (9.81,10.94)	10.06 (9.72,10.39)
2. Post-treatment (day 4 sample) LnRFU means (95% CI) for each production group	11.24 (11.10, 11.38)	10.75 (9.23, 12.27)	10.74 (10.39, 11.08)
3. Number of positive samples based on ROC analysis ^3^ a. Pre-treatment pen oral fluid samples b. Post-treatment pen oral fluid samples (lnRFU mean, 95% CI) ^1^	0/3 3/3 11.24 (11.10, 11.38)	0/3 1/3 11.45	0/6 4/6 10.93 (10.65, 11.21)

^1^ Fluorescence reported as log-transformed relative fluorescence units (ln(RFUs)). ^2^ Red food coloring (McCormick & Co., Inc., Hunt Valley, MD, USA) at a 1:1 dilution with a sucrose solution. ^3^ Receiver operating characteristic (ROC) analysis (pROC package v1.18.5) performed on the aggregate data, with Youdin’s J used as the cutoff.

## Data Availability

The original contributions presented in this study are included in the article. Further inquiries can be directed to the corresponding author.
